# Assessing the tolerance to room temperature and viability of freeze-dried mice spermatozoa over long-term storage at room temperature under vacuum

**DOI:** 10.1038/s41598-018-28896-8

**Published:** 2018-07-13

**Authors:** Yuko Kamada, Sayaka Wakayama, Ikue Shibasaki, Daiyu Ito, Satoshi Kamimura, Masatoshi Ooga, Teruhiko Wakayama

**Affiliations:** 1 Faculty of Life and Environmental Sciences, University of Yamanashi, Yamanashi, 400-8510 Japan; 2 Advanced Biotechnology Centre, University of Yamanashi, Yamanashi, 400-8510 Japan

## Abstract

Freeze-drying has been frequently used to preserve food and microorganisms at room temperature (RT) for extended periods of time; however, its application to mammalian species is difficult. Here, we developed a method to prolong the stability of freeze-dried (FD) mice spermatozoa at RT for more than one year without using any cryoprotectant agents. Our data showed that maintaining a vacuum in ampoules is critical to ensuring the viability of FD spermatozoa, as the stability of spermatozoa DNA increased when imperfectly vacuumed ampoules were detected using a non-destructive test and eliminated. Finally a large number of healthy offspring were obtained from mice oocytes fertilized with FD spermatozoa stored at RT for more than one year. Although the birth rate from three-month stored spermatozoa was lower than that from one-day stored spermatozoa, no further reduction was observed even in one-year stored spermatozoa. Therefore, FD spermatozoa preserved in this study were highly tolerant to warm temperatures. This method of storage shows a great potential for the preservation of genetic resources of mammalian species, such as genetically-modified mouse strains, without the use of electric power.

## Introduction

Preservation of spermatozoa plays an important role in the treatment of infertility, domestic production of animals, preservation of genetically-modified mouse strains, and protection of endangered or extinct species^1^. Generally, cryopreservation of spermatozoa involves mixing the spermatozoa with a cryoprotectant of choice and freezing these cryoprotectant-immersed spermatozoa in liquid nitrogen (LN_2_). Spermatozoa preserved using this method retain their motility after thawing and can be used to produce healthy offspring using artificial insemination or *in vitro* fertilization. However, the use of LN_2_ for freezing spermatozoa may not be ideal for many reasons: LN_2_ must be handled very carefully because of its extremely low temperature of −196 °C and danger of suffocation; transporting spermatozoa in LN_2_ can be problematic, as it is a liquid; and LN_2_ may not be readily available in some developing countries^2^. Most importantly, the use of LN_2_ adds to the cost of spermatozoa preservation, which is incumbent on the couples seeking infertility treatment. Spermatozoa can also be preserved in an ultra-deep freezer at −150 °C; however, this maintenance is highly expensive because of uninterrupted use of electric power.

By contrast, freeze-drying is a reliable and commonly used technique in many countries. Freeze-drying is a dehydration process typically used to preserve live, perishable materials such as food or microorganisms for long-term storage at room temperature (RT) without the use of any preservatives. Freeze-drying has also been successfully used for the preservation of mammalian spermatozoa. We demonstrated for the first time that spermatozoa can be freeze-dried (FD) and stored for three months at 4 °C or for one month at RT without losing their reproductive potential^3^. Since then, this method has been used not only for mouse spermatozoa but also for spermatozoa of other animals such as rats and rabbits^2,4,5^. Notably, FD spermatozoa can be preserved permanently when stored in an ordinary freezer^6–9^.

The preservation of FD spermatozoa at RT enables cost-effective transportation of spermatozoa^3,10^. Benefiting from this advantage, we recently sent FD spermatozoa to the International Space Station to examine the effect of space radiation on the integrity of sperm DNA^11^. This travel from earth to the International Space Station and back was possible because ampoules of FD spermatozoa could be conveniently stored at RT; therefore, installation of an ultra-deep freezer or another form of special protection in the rocket was not needed. Despite its advantages the method of freeze-drying spermatozoa has not yet been utilized because of its poor reliability for long-term preservation at RT. For example, freeze-drying itself has been previously shown to damage the DNA of spermatozoa^12^. Recently, other sperm drying methods, such as evaporative drying and microwave drying, have been reported^13–16^. Although these methods do not require the expensive freeze-drying machine, they require special storage. Therefore, the utility of these methods and their superiority over the freeze-drying method has yet to be demonstrated.

In this study, we investigated the reasons underlying the loss of developmental potential of FD spermatozoa at RT with increase in the length of the preservation period. We developed techniques to identify ampoules with imperfect vacuum using the Tesla coil leak detector and to minimize the amount of air trapped in ampoules after vacuuming using moisture-absorbing agents. FD spermatozoa maintained under a tight vacuum could be stored at RT without any cryoprotectant for more than one year, and were used to produce a large number of healthy offspring. This preservation methods do not require any electric power or other expensive inputs because the use of a desk drawer is sufficient. Therefore, it will be an important tool not only for assisting human reproduction in cases of infertility but also for preserving the genetic diversity of mammalian species in the event of an earthly disaster.

## Results

### Assessment of the quality of FD spermatozoa stored at RT

**Figure 1:**
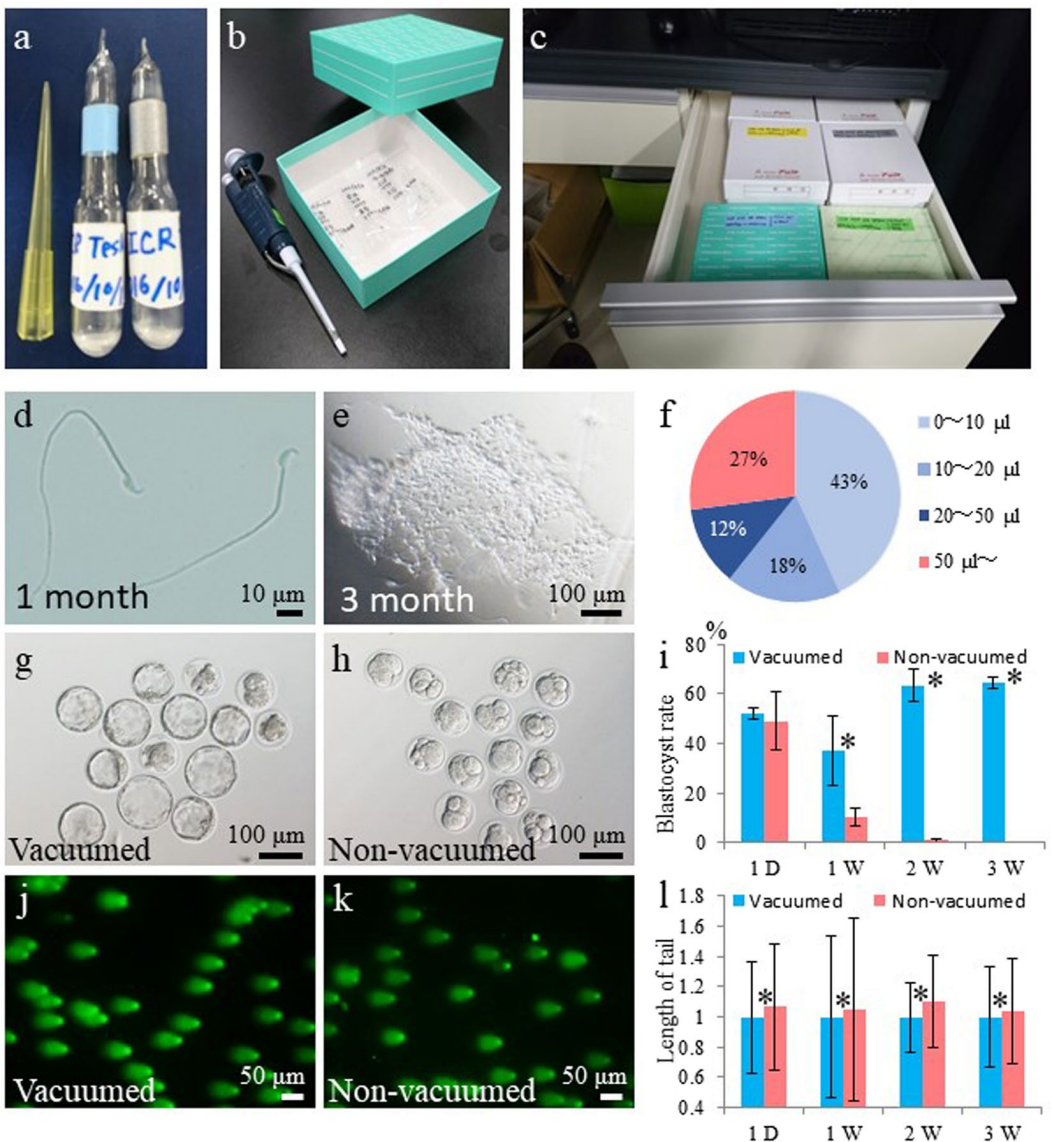
Preservation of FD spermatozoa at room temperature and effect of air in ampoules. (**a**) Ampoules of FD spermatozoa. (**b**) Paper boxes used to store ampoules. (**c**) Paper boxes containing spermatozoa ampoules were stored in a desk drawer for up to 16 months without the use of any protective chemicals. (**d**) Single spermatozoa recovered after rehydration of ampoules stored at RT for 1 month. (**e**) Spermatozoa aggregates that failed to rehydrate after being stored at RT for more than 3 months. (**f**) Pie-chart distribution of per cent ampoules (*n* = 229) containing variable amounts of trapped air. Fertilized embryos (in culture for 4 days) obtained from FD spermatozoa stored in (**g**) vacuumed ampoules or (**h**) non-vacuumed ampoules at RT for 2 weeks. (**i**) The rate of blastocyst development from embryos obtained from FD spermatozoa stored in vacuumed or non-vacuumed ampoules for up to 3 weeks. Comet assay of FD spermatozoa stored in (**j**) vacuumed or (**k**) non-vacuumed ampoules. (**l**) Comparison of comet tail lengths of FD spermatozoa stored in vacuumed or non-vacuumed ampoules. Lengths of comet tails were standardized against mean lengths of vacuumed spermatozoa for each storage period. Asterisk denotes statistically significant differences between samples (*P* < 0.05).

Ampoules containing FD spermatozoa collected from the ICR strain were placed in paper boxes and stored in a desk drawer (Fig. 1a–c). To understand the decline in the fertility of FD spermatozoa over time, we evaluated spermatozoa morphology after 1, 3, and 6 months of storage at RT. Ampoules were rehydrated, and the morphology of FD spermatozoa was observed. After one month of storage, spermatozoa appeared normal and individual spermatozoa could be collected from all ampoules (Fig. 1d). However, after three and six months of storage, contents of 15% and 26% ampoules, respectively, did not dissolve upon rehydration, and compact aggregates of spermatozoa were observed (Fig. 1e and Supplemental Fig. 1a).

### Detection of trapped air in ampoules

We hypothesized that aggregation of FD spermatozoa in three- and six-month-old samples probably occurred due to incomplete vacuuming of ampoules and the presence of trace amounts of moisture and/or oxygen. To test this hypothesis, each ampoule was broken under water (Supplemental Fig. 1b). Air bubbles escaping from all ampoules were observed without exception (Supplemental Fig. 1c,d), which was indicative of trapped air. However, the volume of trapped air varied among ampoules from <10 μl (approximately 1% of ampoule volume) in 43% of ampoules to >50 μl in 27% of ampoules (Fig. 1f). Overall, these data indicated that the vacuuming of ampoules was incomplete.

### Developmental potential of FD spermatozoa stored in air

In preliminary experiments, differences were detected in the rate of full-term development between spermatozoa harvested from ampoules prepared at the same time from the same male (Supplemental Table 1). To determine whether these differences could be explained by the amount of residual air trapped in each ampoule following incomplete vacuuming, ampoules of FD spermatozoa were prepared with or without vacuuming and stored at RT for up to three weeks. Data revealed that the rate of development of zygotes fertilized with FD spermatozoa to the blastocyst stage in non-vacuumed ampoules was lower than that in vacuumed ampoules (Fig. 1g–i and Supplemental Fig. 2; Supplemental Table 2). These differences were apparent after one day of storage at RT and were statistically significant after one week, with zero embryos developing after three weeks of storage at RT (Fig. 1i). The DNA integrity of FD spermatozoa of vacuumed and non-vacuumed ampoules was examined using the comet assay (Fig. 1j–l). FD spermatozoa in non-vacuumed ampoules showed significantly higher DNA damage than those in vacuumed ampoules even after one day of storage (Fig. 1l and Supplemental Table 3). These results suggest that the comet assay is capable of detecting DNA damage even among reproductively competent spermatozoa (see one-day data in Fig. 1i,l). By contrast, when comparing the competency of three-week-old spermatozoa, blastocysts could be obtained only from vacuumed spermatozoa (Fig. 1i); however, comet assay failed to detect such a large difference between vacuumed and non-vacuumed spermatozoa (Fig. 1l).

### Detection of trapped air in ampoules using Tesla coil leak detector

**Figure 2:**
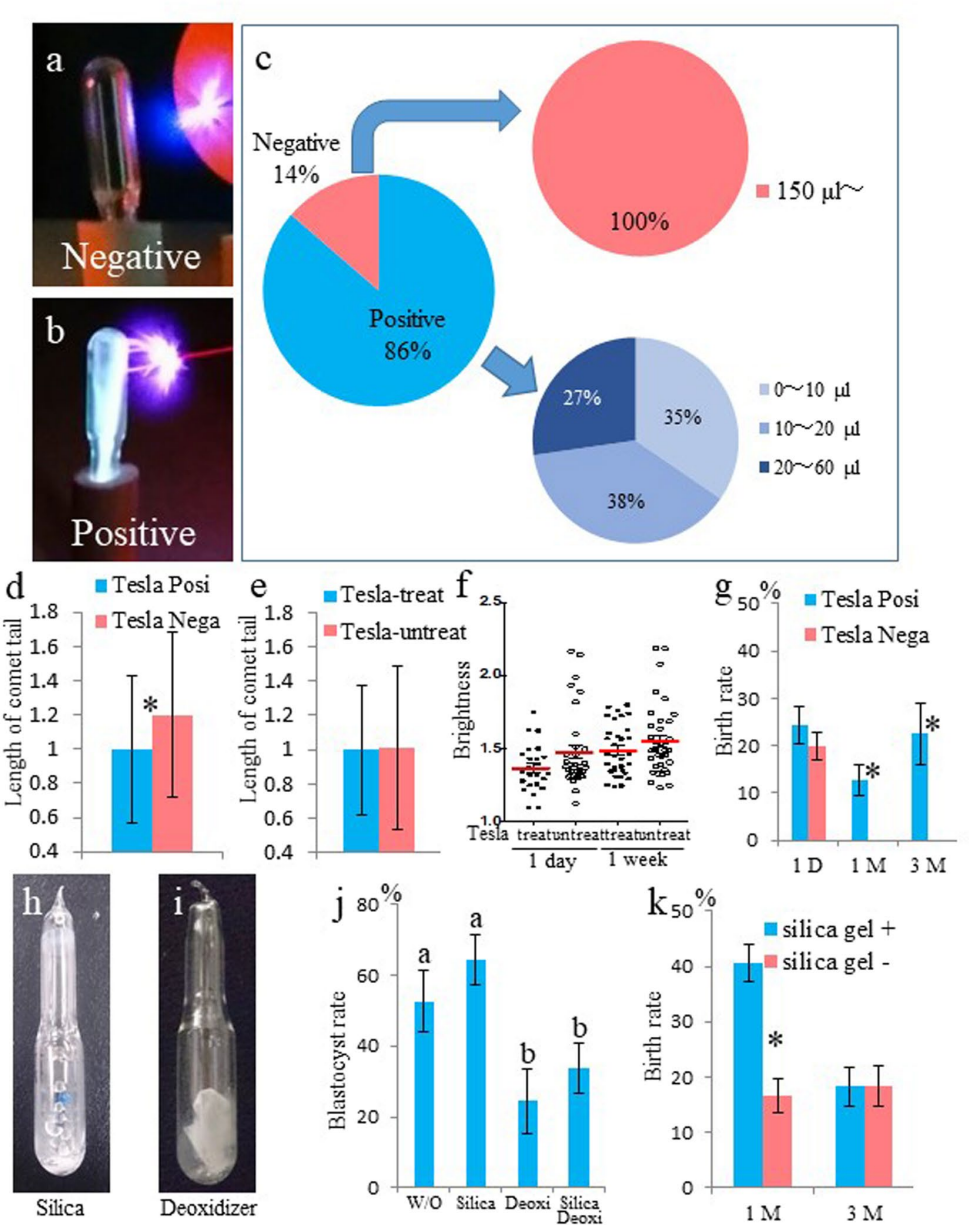
Detection and measurement of trapped air in ampoules using Tesla coil leak detector and air absorbing agents. (**a**) Tesla-negative ampoule containing trapped air. (**b**) Tesla-positive ampoule containing no or minimal air. (**c**) Percentage of Tesla-positive and Tesla-negative ampoules out of 198 and the relative proportion (%) of ampoules containing variable amounts of trapped air. Comparison of comet tail lengths of FD spermatozoa stored in (**d**) Tesla-positive and Tesla-negative ampoules, and (**e**) Tesla-treated and Tesla-untreated ampoules. (**f**) Result of anti-gamma-H2AX antibody staining. Brightness plots of male pronuclei obtained from fertilization of oocytes with 1-day- or 1-week-old spermatozoa stored at RT in Tesla-treated or Tesla-untreated ampoules. (**g**) Full-term development of embryos obtained from spermatozoa stored in Tesla-positive or Tesla-negative ampoules stored at RT for up to 3 months. Ampoule of FD spermatozoa with (**h**) silica gel or (**i**) deoxidizer. (**j**) The rate of blastocyst development from embryos derived from FD spermatozoa stored at RT for 3 weeks without any agent (W/O), with silica gel (Silica), deoxidizer (Deoxi), or both (Silica Deoxi). Different letters indicate statistically significant differences (*P* < 0.05). (**k**) Full-term development of embryos derived from FD spermatozoa stored at RT for up to 3 months with or without silica gel. Asterisk or different letters indicates statistically significant differences between samples (*P* < 0.05).

Breaking the ampoules under water to measure the amount of residual air is a destructive assay, as those spermatozoa cannot be used for further experimentation. To overcome this problem, we developed a nondestructive assay using a Tesla coil leak detector, which allows the detection of trapped air without breaking the ampoules. The Tesla detector is a device which is relatively small and easy to handle (Supplemental Fig. 3). When the tip of the Tesla coil is brought near the ampoule, the tip will spark around the glass. If a lot of air is trapped inside the ampoule, the air cannot be ionized (Fig. 2a). However, if the ampoule contains only a small amount of residual air, its ionization produces a spark inside the ampoule (Fig. 2b).

We tested 198 ampoules containing FD spermatozoa using the Tesla coil leak detector. Approximately 86% of the ampoules were tested as Tesla positive (Fig. 2c). One ampoule showed intermediate color but was determined as Tesla negative. To confirm the results obtained with the Tesla detector, all examined ampoules were opened under water, and the amount of trapped air was measured. All Tesla-negative ampoules (27) contained >150 μl air, whereas all Tesla-positive ampoules (171) contained <60 µl air (Fig. 2c). The degree of DNA damage of FD spermatozoa was higher in Tesla-negative ampoules than in Tesla-positive ampoules, based on comet assay results (Fig. 2d and Supplemental Table 4).

### Effect of the Tesla detector test on the quality of FD spermatozoa

Because the Tesla coil generates high voltage, low current, and high frequency AC electricity, we determined whether the use of Tesla detector affected the DNA quality of FD spermatozoa using the comet assay and gamma-H2AX assay. The lengths of comet tails did not differ between Tesla-treated and Tesla-untreated spermatozoa (Fig. 2e), which suggested that the Tesla detector did not damage the DNA of FD spermatozoa. The injection of Tesla-treated spermatozoa into oocytes resulted in fertilization and normal-appearing pronuclei, similar to the control. The resulting zygotes were immunostained with anti-gamma-H2AX antibody. Several foci were detected in the male pronuclei (Supplemental Fig. 4). Because it was difficult to count the number of foci within the pronuclei, we measured the brightness of the whole male pronucleus and subtracted it from that of the zygote cytoplasm. No significant differences were detected between the Tesla-treated and Tesla-untreated spermatozoa, irrespective of the storage period (Fig. 2f and Supplemental Table 5).

Fertilization of oocytes with one-day-old FD spermatozoa harvested from both Tesla-positive and Tesla-negative ampoules produced live offspring (Fig. 2g). However, fertilization with FD spermatozoa stored at RT for one to three months resulted in live offspring only when the spermatozoa were obtained from Tesla-treated ampoules, the success rate of which was comparable with that obtained using one-day-old spermatozoa (Fig. 2g and Supplemental Table 6). Although one intestinal hernia pup (Supplemental Fig. 5a) was obtained from using three-month-old Tesla-treated spermatozoa, it was unclear whether this was the effect of Tesla treatment or just a chance occurrence.

### Effect of silica gel and deoxidizer on FD spermatozoa preservation

To ensure complete removal of moisture and oxygen from vacuumed ampoules, the effect of silica gel and deoxidizer on the quality of FD spermatozoa was examined (Fig. 2h,i). The rate of development to the blastocyst stage was slightly higher with spermatozoa obtained from three-week-old ampoules containing silica gel than from the control (Fig. 2j and Supplemental Table 7). On the other hand, the rate of development to the blastocyst stage decreased significantly with spermatozoa stored in ampoules containing deoxidizer alone or in combination with silica gel. Therefore, we decided to use only silica gel for all subsequent experiments. However, no differences were detected in the rate of development to the blastocyst stage between spermatozoa stored in ampoules with or without silica gel for one and three months, respectively (Supplemental Table 8). After embryo transfer, although significantly higher birth rate was obtained from silica gel-containing ampoules after one month of preservation, this rate was reduced and became comparable to the control after three months of preservation (Fig. 2k and Supplemental Table 9). Although the silica gel retained its blue color (active state) inside the ampoules even after three months, it is unclear why it was able to preserve FD spermatozoa only for one month.

### Full-term development of zygote fertilized with >1-year-old FD spermatozoa

**Figure 3:**
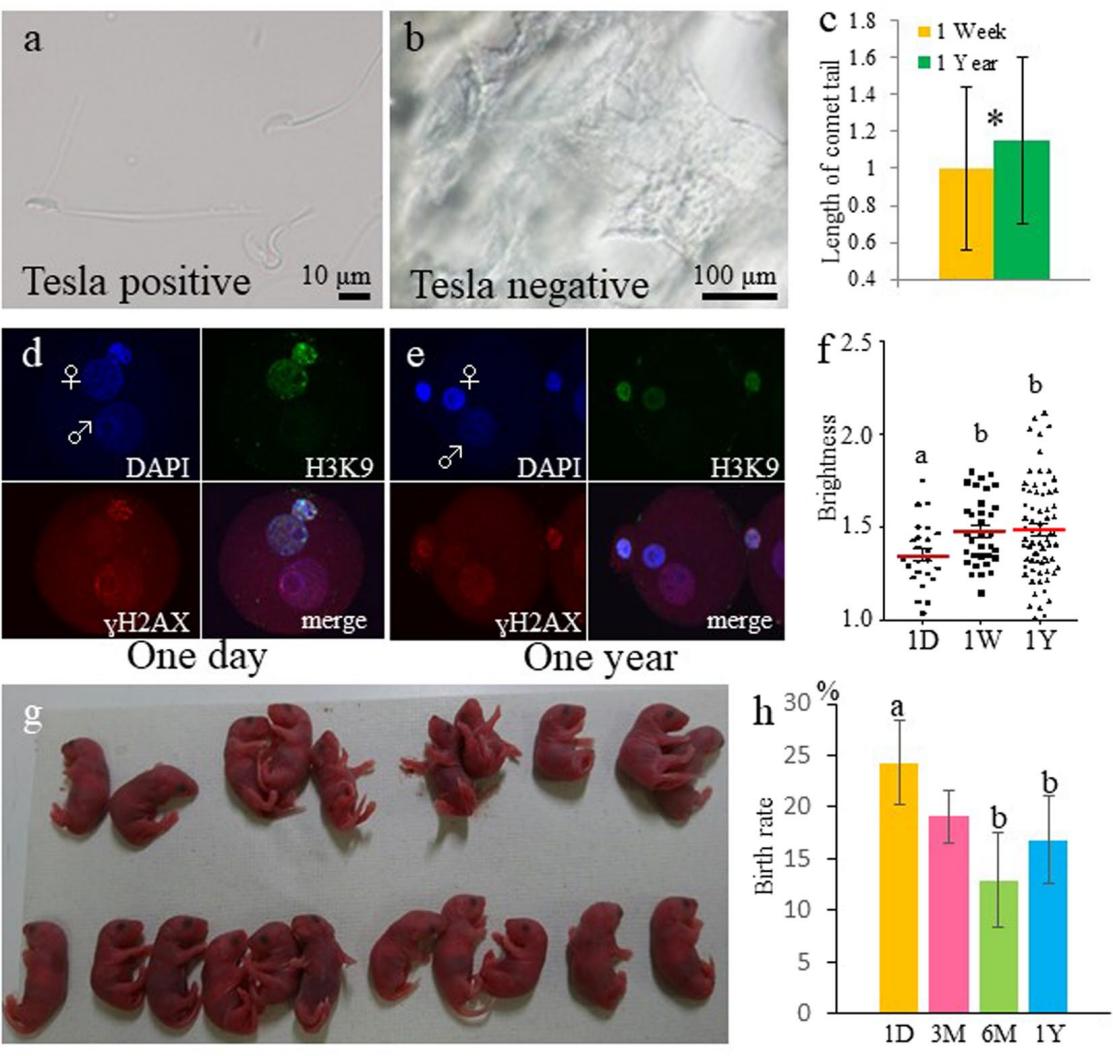
Production of healthy offspring from FD spermatozoa stored at RT for 1 year. (**a**) Single spermatozoa were collected from Tesla-positive ampoules after rehydration. (**b**) Spermatozoa aggregates collected from Tesla-negative ampoules after rehydration. (**c**) Comparison of comet tail lengths of FD spermatozoa stored at RT for less than 1 week or 1 year. Asterisk indicates statistically significant differences between samples at *P* < 0.05. Zygotes derived from FD spermatozoa stored at RT for (**d**) 1 day or (**e**) 1 year and stained with anti-gamma-H2AX antibody. Images show male and female pronuclei stained with DAPI in blue (upper left), female pronuclei labeled with anti-H3K9 me2 antibody in green (upper right), gamma-H2AX signals indicating double-stranded DNA breaks in red (lower left), and merged images (lower right). (**f**) The brightness of each male pronucleus was plotted. The brightness of male pronucleus in (**d**) was 1.44 and in (**e**) was 1.50. Different letters indicate statistically significant differences at *P* < 0.05. (**g**) Live offspring derived from FD spermatozoa stored at RT for 1 year. (**h**) The rate of full-term development of embryos derived from FD spermatozoa preserved for 1 day, 3, 6, and 12 months at RT. The 1 day data and Fig. 2g, 1 day, Tesla Posi. is same. Different letters indicates statistically significant differences between samples (*P* < 0.05).

Rehydration of FD spermatozoa stored at RT for more than one year yielded free-floating, individual spermatozoa from Tesla-positive ampoules (Fig. 3a) and aggregates of spermatozoa from Tesla-negative ampoules (Fig. 3b). Therefore, only Tesla-positive ampoules were used for this experiment. FD spermatozoa stored at RT for one year showed significantly longer comet tails than those stored at RT for one week (Fig. 3c and Supplemental Table 10), suggesting that extended storage at RT damages sperm DNA. Similarly, male pronuclei derived from one-year-old FD spermatozoa showed brighter staining with anti-gamma-H2AX antibody than from those derived from one-day-old FD spermatozoa (Fig. 3d–f). However, no significant differences were detected between the staining intensity of pronuclei derived from one-week- vs. one-year-old FD spermatozoa stored at RT (Supplemental Table 11). Notably, even when one-day-old FD were used, male pronuclei showed brighter staining than female pronuclei, suggesting that DNA of spermatozoa was damaged by FD treatment per se. Whereas comet assay showed significant differences between one-week- and one-year-old FD spermatozoa (Fig. 3c), the gamma-H2AX assay showed no differences (Fig. 3f).

Next, we examined the full-term developmental potential of FD spermatozoa stored at RT for longer periods. Approximately 80% of zygotes produced from the fertilization of oocytes with 3-, 6-, 12- and 16-month-old FD spermatozoa developed to the two-cell stage. On average, 17% of embryos produced from 12- and 16-month-old FD spermatozoa generated viable offspring after being transferred into recipient females (Table 1 and Supplemental Table 12 and Fig. 3g); these numbers were comparable with those obtained from three-month-old (19%) and six-month-old (13%) spermatozoa (Fig. 3h). Except for one pup that was stillborn (Supplemental Fig. 5b), all other pups (99.2%) were alive and showed normal phenotype. The sex ratio of pups obtained from 12-month-old spermatozoa was normal (male: 42%; female: 58%). These pups were grown to adults. Mating between randomly-selected male and female mice produced offspring (Supplemental Table 13), which demonstrated the normal fertility of mice produced from 12-month-old FD spermatozoa.

**Table 1:** Full-term development of embryos derived from FD sperm preserved for 1 year at RT.

Storage periods	No. of oocytes surviving after ICSI	No. (%) of fertilised embryos	No. (%) of two-cell embryos at 24 h	No. of transferred embryos (no. of recipients)	No. (%) [min–max] of offspring**	Mean body weight (g)
Fresh control	37	35 (94.6)	33 (94.3)	33 (3)	19 (57.6) [50.0–70.0]	1.63 ± 0.21
1 day*	275	270 (98.2)	214 (79.2)	214 (9)	52 (24.3)a [9.5–36.4]	1.77 ± 0.18
3 month	344	323 (93.9)	298 (92.3)	298 (13)	57 (19.1) [12.1–31.3]	1.86 ± 0.33
6 month	105	100 (95.2)	85 (85.0)	85 (4)	11 (12.9)b [7.8–33.5]	1.98 ± 0.34
1 year	1259	981 (78.4)	758 (77.3)	758 (27)	127 (16.8)b [0–39.1]	1.85 ± 0.21

^*^This data and Table S6 (1 day, Posi) is same.

^**^Different letters indicates statistically significant differences between samples (*P* < 0.05).

## Discussion

In this study, we demonstrated that healthy offspring, with normal morphology and fertility, could be obtained from FD spermatozoa stored at RT for more than one year. These spermatozoa were sealed in glass ampoules under vacuum, which were placed in paper boxes and stored in a desk drawer at RT without any cryoprotectant or special protection.

The freeze-drying method is commonly used across many countries for the reliable preservation of food over long periods at RT. About 20 years ago, FD mice spermatozoa were used to produce healthy offspring. Recently, other sperm drying methods, such as evaporative drying or microwave drying, have been reported^13–16^. Spermatozoa preserved by evaporative drying in a LiCl sorption jar in the presence of trehalose, as a cryoprotectant, have been used to obtain healthy offspring after storage at ambient temperatures for two years^17^. Although FD spermatozoa can be preserved in easy to handle glass ampoules, the freeze-drying machine is expensive, therefore it was unclear which of the two methods, evaporative drying or freeze-drying, was superior for preserving mammalian spermatozoa at RT^18^.

In this study, we determined that residual air trapped in ampoules is a major factor contributing to the loss of developmental potential of FD spermatozoa within a few months. To minimize air leak into ampoules, we upgraded the equipment used to store FD spermatozoa to new ampoules and new grease. Although this treatment reduced the amount of residual air trapped in many ampoules, some ampoules still continue to trap a relatively large volume of air. However, deoxidizer could not reduce the residual air in ampoules because it contains iron powder as well as water to absorb oxygen and generates heat during the reaction, which may negatively affect the quality of FD spermatozoa.

In addition, we used a Tesla coil leak detector to determine whether ampoules were completely vacuumed. This equipment is generally used to identify defective products, such as semiconductors; however, it has not previously been used for live organisms. In this study, we demonstrated the use of a Tesla detector for the identification of ampoules that were imperfectly vacuumed and contained trapped air. We showed that the spark generated by the Tesla detector did not affect for the quality of FD spermatozoa. Avoiding the use of ampoules with trapped air helped to improve the rate of development to full-term. Although two deformed pups were born from spermatozoa stored in Tesla-treated ampoules, it was unclear whether this phenomenon was caused by Tesla treatment or by long-term preservation at RT.

Cryoprotectants assist in the restoration of life in frozen cells and organisms after thawing or rehydration. For example, Tardigrades can survive under extreme dehydration conditions because of the accumulation of large amounts of trehalose in their bodies which maintains the integrity of cell membranes or organelles^19^. However, it has been reported that cryoprotectants increase the birth rate from dried spermatozoa as well as the success rate of somatic cell cloning^17,18,20–24^, spermatozoa and cells used in these studies were no longer viable following the drying treatment even in the presence of trehalose, and the cell membranes were largely damaged^3^. However, it may be argued that preserving the integrity of the DNA of FD spermatozoa is more important than maintaining the viability of sperm. When DNA damage in FD spermatozoa was examined by gamma-H2AX, the detectable damage did not increase upon longer storage (>1 year) at RT (Fig. 3f) without using any cryoprotectant. The gamma-H2AX assay only examined zygotes fertilized with morphologically normal spermatozoa; therefore, damage could not be detected solely by comparing comet assay, which examined spermatozoa without selection (Fig. 3c). Results of this study and previous reports^17,18,20–24^ suggest that cryoprotectant protect the cell organelle and not the DNA from mechanical stress. Therefore, if our method (maintaining vacuum in ampoules) is combined with using cryoprotectant, morphological integrity as well as DNA integrity of FD spermatozoa will be protected, thereby increasing the success rate of the offspring from those spermatozoa.

A recent study in reproductive biotechnology has demonstrated that transferring the nucleus or DNA from dead spermatozoa or cells into oocytes can be used to regenerate life. For example, healthy offspring have been obtained using dead spermatozoa or somatic cells harvested from >10-year-old frozen cadavers^25–27^. Therefore, if DNA integrity of FD spermatozoa is preserved at RT under complete vacuum and DNase-free environment^2^, it may be possible to generate life from these spermatozoa using reproductive biotechnology tools.

Recently, seeds of several plant species have been preserved in the Svalbard Global Seed Vault to maintain the genetic diversity of plants. This facility can operate continually even without electric power, in event of a disaster occurring on earth^28^. Although it is highly important to maintain the genetic diversity in mammalian species, spermatozoa or oocyte/embryos cannot be preserved in the event of such a disaster because an uninterrupted supply of LN_2_ is required for their preservation. According to our previous suggestion, underground tunnels on the moon, such as lunar lava tubes^29,30^, would serve as an ideal storage for the permanent preservation of mammalian gametes, as the tunnels are very cold and would be completely isolated from any earthly disasters^11^. However, with current technology, this method is not realistic and is highly expensive. Based on data presented in this study, the freeze-drying method has a high potential to preserve mammalian spermatozoa at RT for long periods of time without the use of cryoprotectants or special storage. Moreover, freeze-drying of gametes can be used as an economical means to treat infertility and preserve the genetic resources of species on the verge of extinction.

## Materials and Methods

### Animals

ICR and BDF1 (C57BL/6N × DBA/2) female mice (8–10 weeks of age) were obtained from SLC Inc. (Hamamatsu, Japan). The surrogate pseudopregnant ICR females, used as recipients of embryos, were mated with vasectomized ICR males, whose sterility had been previously demonstrated. On the day of the experiment or after finishing all experiments, mice were euthanized by CO_2_ inhalation or cervical dislocation and used for experiments. All animal experiments followed the Guide for the Care and Use of Laboratory Animals and were approved by the Institutional Committee of Laboratory Animal Experimentation of the University of Yamanashi.

### Media

HEPES-CZB medium^31^ and CZB^32^ were used for oocyte/embryo manipulation and incubation in 5% CO_2_ at 37 °C, respectively. HTF medium^33^ was used for freeze-drying spermatozoa.

### Preparation of FD spermatozoa

Both epididymides were collected from male mice, and ducts were cut with a pair of sharp scissors. A few drops of the dense spermatozoa mass were then placed into a centrifuge tube containing 2 ml HTF medium and incubated for 30 min at 37 °C in 5% CO_2_. The concentration of spermatozoa was measured, and 50 μl aliquots of the spermatozoa suspension were dispensed into glass ampoules. The ampoules were flash-frozen in LN_2_ and FD using a FDU-2200 freeze dryer (EYELA, Tokyo, Japan). The cork of the freeze dryer was opened for at least 3 h until all samples were completely dry. After drying, ampoules were sealed by melting the ampoule necks using a gas burner under vacuum, as described previously^11^ (Fig. 1a).

### Preservation of FD spermatozoa at RT

All ampoules were placed in small plastic bags and then in paper boxes (Fig. 1b) and stored in a desk drawer at RT (15 °C–25 °C) (Fig. 1c) until further use. No additional protective measures were taken for the preservation of spermatozoa.

### Measurement of air trapped in ampoules

To measure the amount of air trapped in the imperfectly vacuumed ampoules, each ampoule was broken under water (Supplemental Fig. 1). A small bubble of air released from the ampoule was captured using a 100-μl glass capillary and measured.

### Detection of trapped air in ampoules using the Tesla coil leak detector

Ampoules containing air were identified using a Tesla coil leak detector (Sanko Electronic Laboratory Co., Ltd.), according to the manufacturer’s instructions. Briefly, ampoules were exposed to the Tesla detector in the dark. When the Tesla coil tip with minimum power was brought near the ampoule containing air, the tip sparked around the glass and ionized the low-pressure gas inside the ampoule. Therefore, ampoules that turned bright with the Tesla detector were determined as vacuumed.

### Absorption of residual air in ampoules

To remove all traces of moisture and oxygen from ampoules, silica gel (Staclean Protec, Sakurai Co. Ltd., Japan), deoxidizer (A-750HS, ISO Inc., Japan), or both were inserted into ampoules just prior to the end of freeze-drying. Ampoules were then subjected to vacuum for at least an additional hour and sealed.

### Oocyte preparation

Female mice were superovulated by the injection of 5 IU of equine chorionic gonadotropin, followed by 5 IU of human chorionic gonadotropin (hCG) after 48 h. Cumulus-oocyte complexes (COCs) were collected from the oviducts of females 14–16 h later and moved to a Falcon dish containing HEPES-CZB media. To disperse the cumulus, COCs were transferred into a 50-μl droplet of HEPES-CZB medium containing 0.1% bovine testicular hyaluronidase for 3 min. Cumulus-free oocytes were washed twice and moved to a 20 μl droplet of CZB for culture.

### Intracytoplasmic spermatozoa injection and embryo transfer

Intracytoplasmic spermatozoa injection (ICSI) was performed as described previously^31^. Just before starting ICSI, the neck of an ampoule was punctured and 50 μl of sterile distilled water was immediately added and mixed with a pipette. For microinjection of spermatozoa, 1–2 μl of spermatozoa suspension was moved directly to the injection chamber. The spermatozoa suspension was replaced every 30 min during the ICSI procedure. Application of several piezo pulses separated the spermatozoa head from the tail, and the head was then injected into the oocyte. The oocytes that survived ICSI were incubated in CZB medium at 37 °C with 5% CO_2_. Pronucleus formation was checked at 6 h after ICSI. Embryos at the two-cell stage were transferred to a day 0.5 pseudopregnant mouse that had been mated with a vasectomized male the night before transfer. Six to ten embryos were transferred into each oviduct. At day 18.5 of gestation, offspring were delivered by cesarean section and allowed to mature. The remaining unused embryos were cultured for up to four days to evaluate their potential for developing into blastocysts.

### Analysis and scoring of comet slides

Spermatozoa DNA damage, potentially caused by single- and double-stranded breaks^34^, was measured using the CometAssay® Kit (Trevigen, MD, USA), according to the manufacturer’s instructions. Briefly, spermatozoa specimens were collected from ampoules immediately after opening and were rehydrated in water. Specimen and its counterpart were mounted on same slide, and 100–300 spermatozoa heads on each slide were analyzed by electrophoresis. To standardize the results across different periods and conditions under which the spermatozoa were stored, the length of each DNA comet tail was divided by the mean length of the one-side results in each experiment. In this comet assay, fresh spermatozoa could not be used as control because this would require a different preparation technique, which would have prevented proper comparison between specimens on the same slide.

### Gamma-H2AX assay

Histone H2AX is one of the H2A variants. The serine at position 139 of H2AX is rapidly phosphorylated within seconds of DNA damage. The phosphorylated form of H2AX, designated as gamma-H2AX, forms foci at sites of DNA damage, which recruits various repair and cell-cycle checkpoint proteins^35^. Therefore, gamma-H2AX foci formation was used as a marker of DNA double-strand breaks in male and female pronuclei, and histone H3K9me2 signals were used to distinguish female pronuclei from male pronuclei. All specimens were fixed 10 h after ICSI and stored in refrigerator until staining. Primary antibodies used for immunostaining zygotes included the anti-phospho-H2AX (Ser139) rabbit polyclonal antibody (1:500; Millipore-Merck, Darmstadt, Germany) and anti-histone H3 (dimethyl K9) mouse monoclonal antibody (1:500; Abcam, Cambridge, UK). The secondary antibodies used were Alexa Fluor 488-labeled goat anti-mouse IgG (1:500; Molecular Probes, Eugene, OR, USA) and Alexa Fluor 568-labeled goat anti-rabbit IgG (1:500; Molecular Probes). DNA was stained with 4′6-diamidino-2-phenylindole (DAPI, 2 μg/ml; Molecular Probes). The brightness of each male pronucleus was measured using ImageJ software and subtracted from the brightness of the zygote cytoplasm.

### Statistical analysis

Results of the comet assay and the gamma-H2AX assay were analyzed using the Wilcoxon–Mann–Whitney nonparametric test. The blastocyst and birth rates were evaluated using chi-squared tests. Statistical significance of differences between variables was determined at *P* < 0.05.

## Electronic supplementary material

Supplementary Figures and Tables
