# Supplementary material for: Assessing the tolerance to room temperature and viability of freeze-dried mice spermatozoa over long-term storage at room temperature under vacuum
Source: Sci Rep. 2018 Jul 13;8:10602. doi: 10.1038/s41598-018-28896-8 (PMC6045625; doi:10.1038/s41598-018-28896-8)
Supplement: Supplementary file 1 — Supplementary Figures and Tables [file 41598_2018_28896_MOESM1_ESM.pdf]

Assessing the tolerance to room temperature and viability of freeze-dried mice spermatozoa over long-term storage at room temperature under vacuum

Yuko Kamada, Sayaka Wakayama, Ikue Shibasaki, Daiyu Ito, Satoshi Kamimura, Masatoshi Ooga and Teruhiko Wakayama

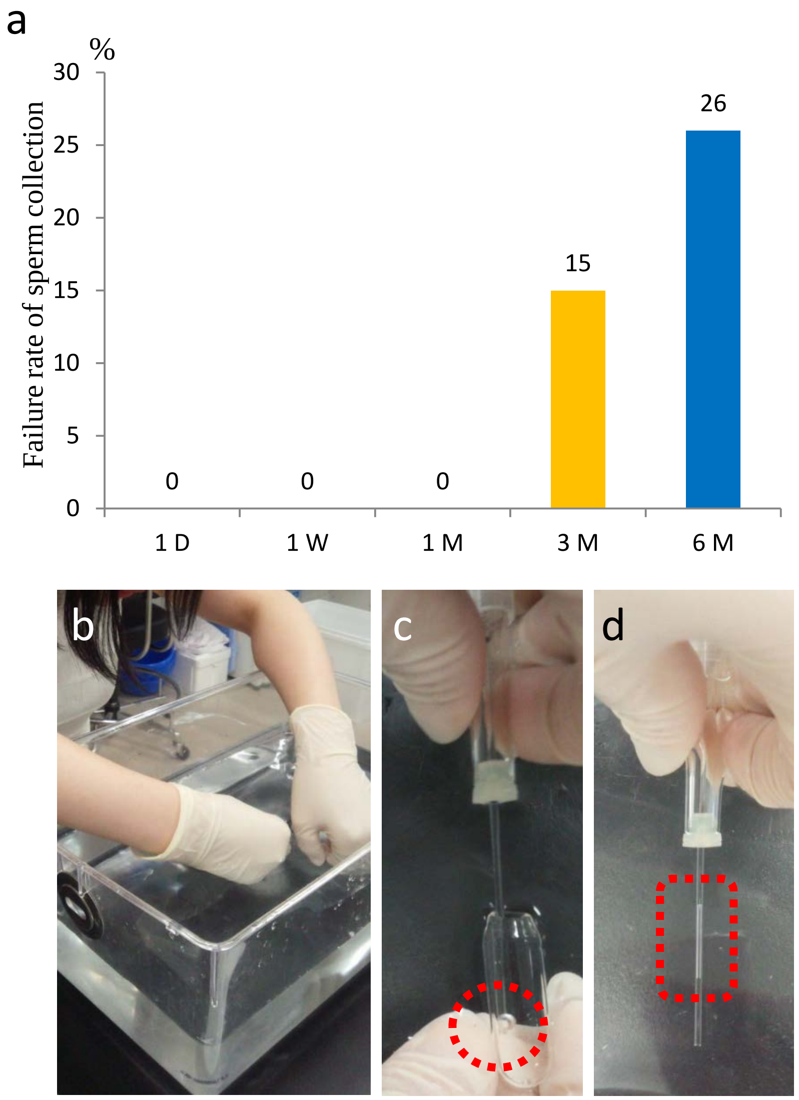

Supplemental Figure 1. Recovery rate of FD spermatozoa after rehydration of ampoules and method used to measure the amount of air trapped in ampoules. (a) The number of FD spermatozoa recovered after rehydration of ampoules stored at RT for up to 6 months. The air trapped in ampoules was measured by breaking the glass ampoules under water (b) and capturing the small air bubble (c) using a glass capillary (d).

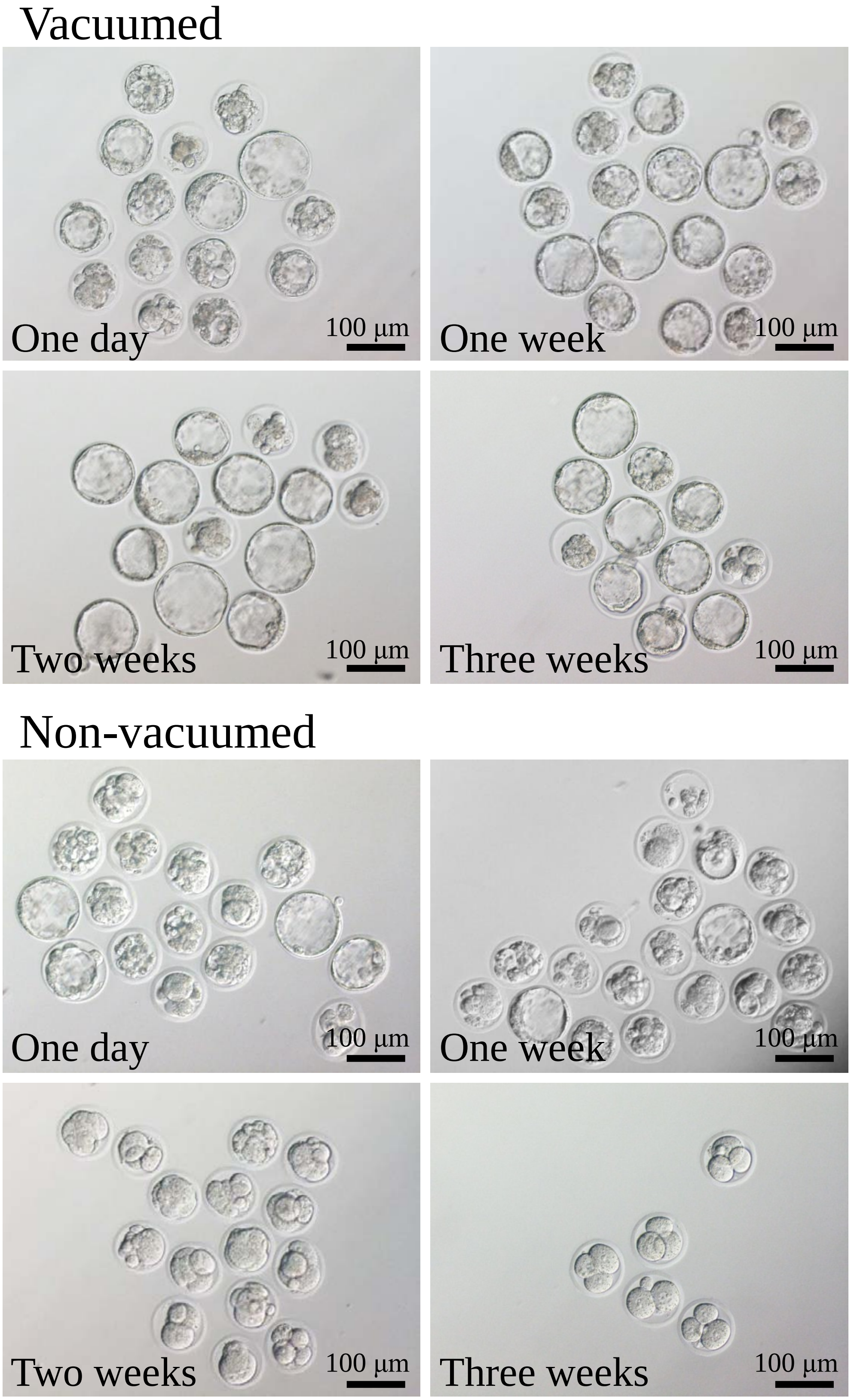

Supplemental Figure 2. Development of embryos derived from FD spermatozoa stored in vacuumed or nonvacuumed ampoules for up to 3 weeks at RT. All images show embryos cultured for 4 days *in vitro*.

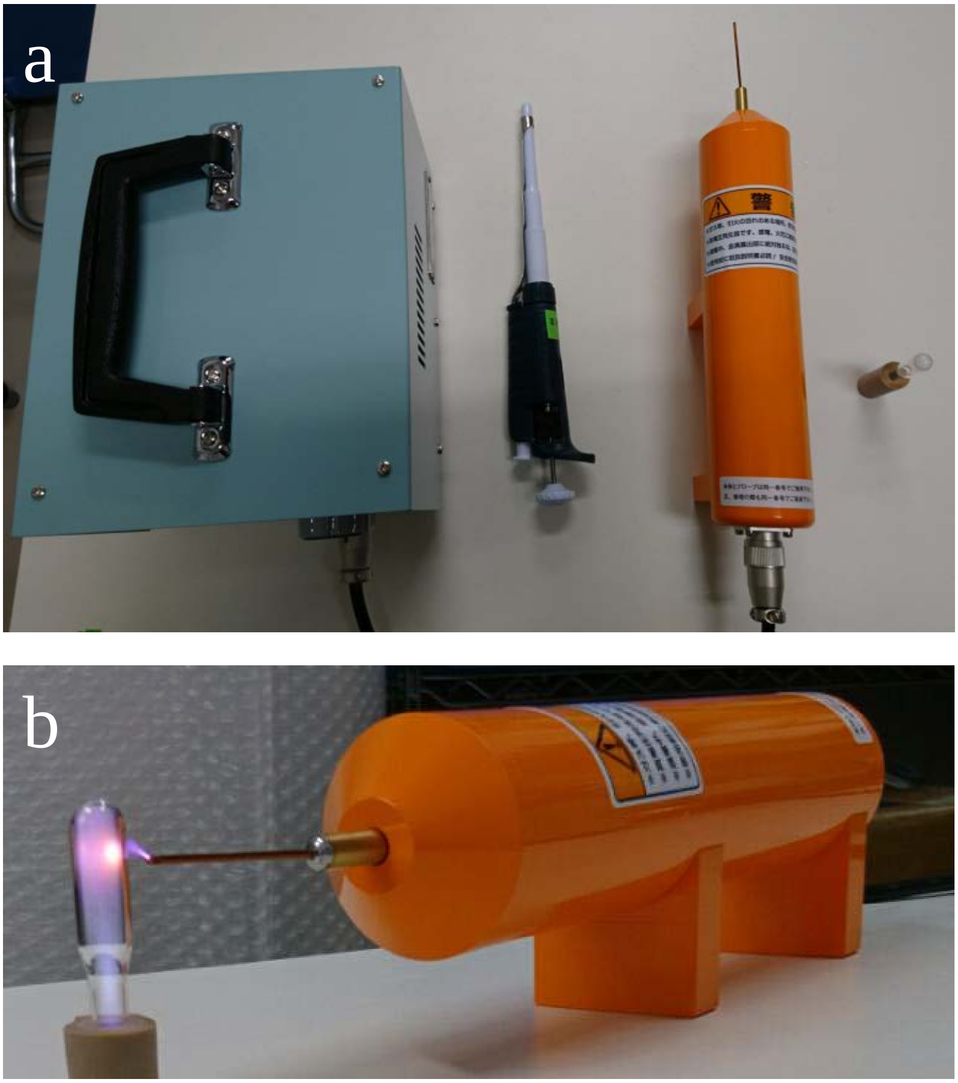

Supplemental Figure 3. Tesla coil leak detector. This is relatively small machine (a) and easy to handle it (b).

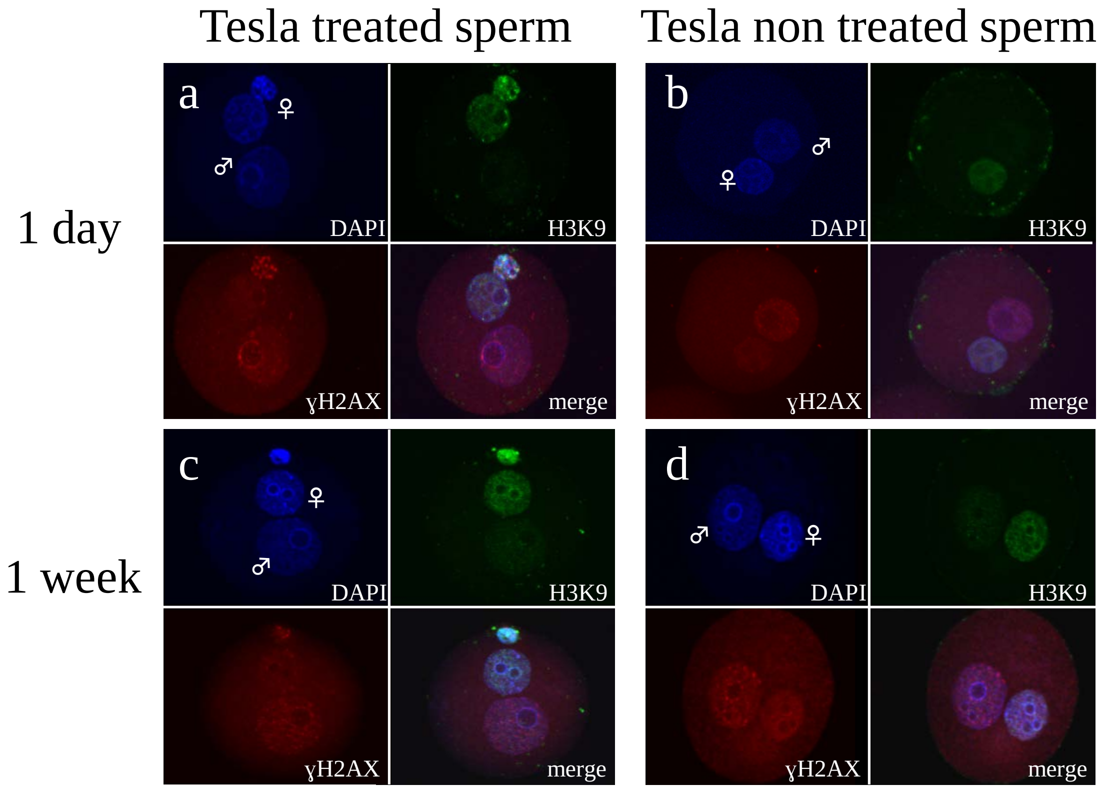

Supplemental Figure 4. Immunostaining of zygotes with anti-gamma-H2AX antibody. Zygotes were derived from FD spermatozoa stored in Tesla-treated or -untreated ampoules at RT for 1 day (a and b) or 1 week (c and d). Images show male and female pronuclei stained with DAPI in blue (upper left), female pronuclei labelled with anti-H3K9 me2 antibody in green (upper right), anti-gamma-H2AX signals indicating double-stranded DNA breaks in red (lower left) and merged images (lower right). The brightness of these pictures (a–d) was 1.44, 1.34, 1.73 and 1.49, respectively.

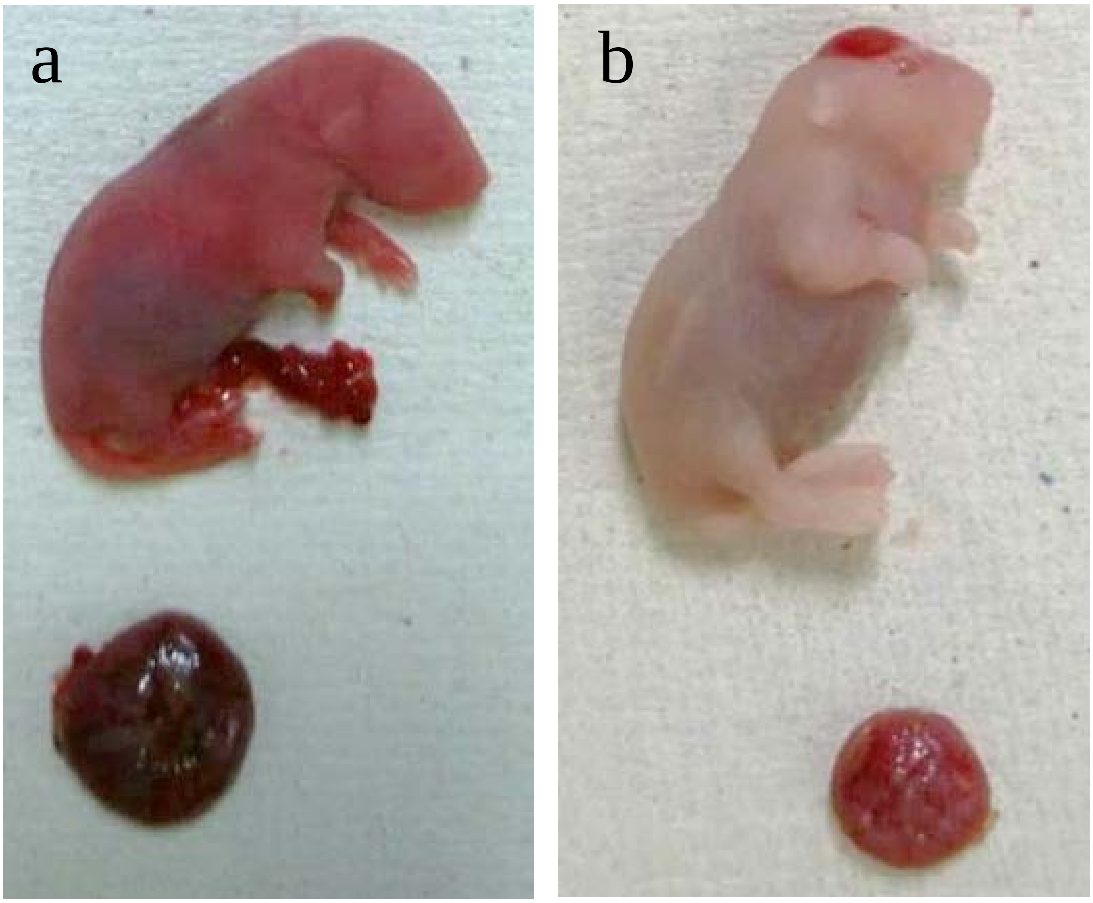

Supplemental Figure 5. Deformed offspring derived from FD spermatozoa preserved at RT. (a) Pup showing intestinal hernia, derived from 3-month-old spermatozoa. (b) Pup without a skull, derived from 1-year-old spermatozoa.

Table S1. Comparison of ampoules between same individual for full-term development after ICSI

Male ID	Ampoule No	No. of oocytes surviving after ICSI	No. (%) of fertilised zygotes	No. (%) of two-cell embryos at 24 h	No. of transferred embryos (no. of recipients)	No. (%) of offspring
A	1	25	25 (100)	23 (92.0)	23 (1)	0 (0)
	2	20	20 (100)	20 (100)	20 (1)	5 (25.0)
	3	18	16 (88.9)	14 (87.5)	14 (1)	4 (28.5)
B	1	25	25 (100)	24 (96.0)	24 (1)	2 (8.3)
	2	30	30 (100)	30 (100)	30 (1)	4 (13.3)
	3	31	30 (96.8)	27 (90.0)	27 (1)	5 (18.5)
C	1	36	33 (91.7)	31 (93.9)	31 (1)	6 (19.4)
	2	34	32 (94.1)	31 (96.9)	31 (1)	7 (22.6)
	3	37	36 (97.3)	35 (97.2)	35 (1)	8 (22.9)

Table S2. Effect of vacuum condition on FD spermatozoa for *in vitro* development following ICSI

Storage periods	Vacuum condition	No. of oocytes surviving after ICSI	No. (%) of fertilised embryos	No. (%) of blastocysts at 96 h
1 day	Vacuumed	111	109 (98.2)	57 (52.3)
	Non-vacuumed	118	110 (93.2)	54 (49.1)
1 week	Vacuumed	168	164 (97.6)	61 (37.2)a
	Non-vacuumed	158	129 (81.6)	13 (10.1)b
2 weeks	Vacuumed	137	134 (97.8)	85 (63.4)a
	Non-vacuumed	129	105 (81.4)	1 (1.0)b
3 weeks	Vacuumed	151	149 (98.2)	96 (64.4)a
	Non-vacuumed	144	84 (58.3)	0 (0)b

Different letters indicate statistically significant differences at $P < 0.05$.

Table S3. Comet assay of FD sperm obtained from vacuumed and nonvacuumed ampoules

Storage periods	Tesla result	No. of examined spermatozoa	Average length of comet tail	SD	*P* value, Kruskal–Wallis test
1 day	Vacuumed	845	1	0.37	
	Non-vacuumed	845	1.07	0.42	< 0.05
1 week	Vacuumed	1568	1	0.54	
	Non-vacuumed	1397	1.05	0.61	< 0.05
2 weeks	Vacuumed	1100	1	0.23	
	Non-vacuumed	943	1.10	0.3	< 0.05
3 weeks	Vacuumed	998	1	0.34	
	Non-vacuumed	839	1.04	0.35	< 0.05

Table S4. Comet assay of FD sperm obtained from Tesla-positive and Tesla-negative ampoules and Tesla-treated and -untreated ampoules

Storage period	Tesla result	No. of examined spermatozoa	Average length of comet tail	SD	*P* value, Kruskal–Wallis test
1 day	Positive	618	1.00	0.43	
	Negative	569	1.2	0.48	< 0.05
	Treated	631	1.01	0.48	
	Non-treated	772	1.00	0.38	> 0.05

Table S5. Brightness of male pronuclei derived from Tesla-treated or -untreated spermatozoa and immunostained with the anti-gamma-H2AX antibody

Storage periods	Tesla treatment	No. of zygotes	Average brightness of male pronucleus	SD	*P* value, Kruskal–Wallis test
1 day	+	25	1.35	0.17	
	-	33	1.47	0.27	> 0.05
7 days	+	33	1.48	0.18	
	-	36	1.53	0.22	> 0.05

Table S6. Full-term development of embryos obtained from Tesla-treated FD spermatozoa

Storage periods	Tesla result	No. of oocytes surviving after ICSI	No. (%) of fertilised embryos	No. (%) of two-cell embryos at 24 h	No. of transferred embryos (no. of recipients)	No. (%) of offspring	Mean body weight (g)
1 day	Posi.*	275	270 (98.2)	214 (79.2)	214 (9)	52 (24.3)	1.77±0.18
	Nega.	269	250 (92.9)	201 (80.4)	201 (8)	40 (19.9)	1.81±0.16
1 month	Posi.	170	165 (97.1)	142 (86.1)	142 (6)	18 (12.7)	1.84±0.29
	Nega.**	-	-	-	-	-	-
3 months	Posi.	149	137 (91.9)	111 (81.0)	111 (6)	25 (22.5)	1.72±0.14
	Nega.**	-	-	-	-	-	-

*: This data and Table 1 (1 day) is same.
**: Any single spermatozoa could be collect from those ampoules.

Table S7. Effect of silica gel and deoxidiser on FD spermatozoa and the resulting embryos obtained via ICSI

Storage periods	Name of agents	No. of oocytes surviving after ICSI	No. (%) of fertilised embryos	No. (%) of blastocysts at 96 h
3 weeks	Without	117	112 (95.7)	59 (52.7)a
	Silica gel	112	110 (98.2)	71 (64.5)a
	Deoxygenate	108	102 (94.4)	25 (24.5)b
	Deoxygenate and silica gel	120	104 (86.7)	35 (33.7)b

Different letters indicate statistically significant differences at $P < 0.05$.

Table S8. Effect of long-term storage of FD spermatozoa in the presence of silica gel on the development of embryos

Storage periods	Silica gel	No. of oocytes surviving after ICSI	No. (%) of fertilised embryos	No. (%) of blastocysts at 96 h
1 month	−	93	67 (72.0)	22 (32.8)
	+	101	99 (98.0)	35 (35.4)
3 months	−	116	93 (80.2)	27 (29.0)
	+	205	203 (99.0)	88 (43.3)

Table S9. Full-term development of embryos derived from FD spermatozoa stored in ampoules containing silica gel

Storage periods	Silica gel	No. of oocytes surviving after ICSI	No. (%) of fertilised embryos	No. (%) of two-cell embryos at 24 h	No. of transferred embryos (no. of recipients)	No. (%) of offspring	Mean body weight (g)
1 month	−	57	57 (100)	54 (94.7)	54 (2)	9 (16.6)a	1.66±0.20
	+	141	136 (96.5)	116 (96.5)	116 (6)	47 (40.5)b	1.91±0.26
3 months	−	172	154 (89.5)	142 (92.2)	142 (5)	26 (18.3)	1.63±0.17
	+	144	142 (98.6)	131 (92.3)	131 (6)	24 (18.3)	1.68±0.15

Table S10. Analysis of FD sperm stored at RT for 1 week or 1 year using comet assay

Storage periods	No. of spermatozoa	Average length of comet tail	SD	*P* value, Kruskal–Wallis test
Within 1 week	1300	1	0.44	
1 year	943	1.15	0.45	< 0.05

Table S11. Brightness of male pronuclei derived from FD spermatozoa stored at RT for 1 day, 1 week or 1 year and immunostained with anti-gamma-H2AX antibody

Storage periods	No. of zygotes	Average brightness of male pronucleus	SD	*P* value, Kruskal–Wallis test
1 day	25	1.35	0.17	
1 week	33	1.48	0.18	< 0.05
1 year	70	1.49	0.26	< 0.05

Table S12. Full-term development of embryos derived from FD sperm preserved for 1 year at RT

Storage periods	Male ID	No. of oocytes surviving after ICSI	No. (%) of fertilised embryos	No. (%) of two-cell embryos at 24 h	No. of transferred embryos (no. of recipients)	No. (%) of offspring	Mean body weight (g)
3 months	2	51	41	33 (80.5)	33 (2)	5 (15.2)	1.78±0.16
	3	33	33	31 (93.9)	31 (2)	9 (29.0)	2.32±0.12
	12	30	28	27 (96.4)	27 (1)	6 (22.2)	1.97±0.09
	13	19	19	18 (94.7)	18 (1)	5 (27.8)	2.23±0.07
	14	33	31	31 (100)	31 (1)	3 (9.7)	1.49±0.08
	15	19	18	16 (88.9)	16 (1)	5 (31.3)	1.88±0.12
	16	36	33	29 (87.9)	29 (1)	5 (17.2)	1.92±0.12
	17	70	70	66 (94.3)	66 (2)	8 (12.1)	1.61±0.29
	18	53	50	47 (94.0)	47 (2)	11 (23.4)	1.54±0.17
6 months	1	64	62	51 (82.3)	51 (2)	4 (7.8)	2.08±0.22
	10*	19	19	17 (89.5)	17 (1)	4 (23.5)	2.22±0.11
	11	22	19	17 (89.5)	17 (1)	3 (17.6)	1.52±0.17
1 year	1	103	81	59 (72.8)	59 (2)	2 (3.4)	2.04±0.22
	2	249	183	175 (95.6)	175 (4)	25 (14.3)	1.93±0.13
	3	70	66	48 (72.7)	48 (2)	17 (35.4)	1.75±0.17
	4	323	252	212 (84.1)	212 (8)	35 (16.5)	1.99±0.20
	5	147	93	63 (67.7)	63 (2)	9 (14.3)	1.94±0.22
	6	99	75	37 (49.3)	37 (2)	0 (0)	-
	8*	49	46	32 (69.6)	32 (2)	10 (31.2)	1.71±0.08
	9*	47	45	27 (60.0)	27 (1)	2 (7.4)	1.59±0.07
	10*	37	34	23 (67.6)	23 (1)	9 (39.1)	1.86±0.18
1 year 4 months	7	128	106	82 (77.4)	82 (3)	18 (22.0)	1.69±0.11

*BDF1 oocytes were used for ICSI.

Table S13. Fertility of offspring derived FD spermatozoa stored for 1 year at RT.

Offspring pair ID*	Birth date of offspring	Delivery date of next generation	Period from birth day of parents and their first delivery day (day)	No. of offspring
1	2017/8/26	2017/10/25	60	15
		2017/12/1	97	5
2	2017/8/28	2017/11/1	64	4
		2017/11/10	73	15
3	2017/9/9	2017/11/16	68	14
		2017/11/20	72	13
4	2017/9/10	2017/11/10	61	13
		2017/11/13	64	15

*One male and two female offspring were randomly selected from each experiment and mated with each other.
